# Autoimmune activation of the GnRH receptor induces insulin resistance independent of obesity in a female rat model

**DOI:** 10.14814/phy2.14672

**Published:** 2020-12-23

**Authors:** Hongliang Li, Gege Zhang, Yankai Guo, Jielin Deng, Hayley Fischer, LaTasha B. Craig, David C. Kem, Xichun Yu

**Affiliations:** ^1^ Section of Endocrinology and Diabetes Department of Medicine University of Oklahoma Health Sciences Center Oklahoma OK USA; ^2^ Cardiac Pacing and Electrophysiology Department The First Affiliated Hospital of Xinjiang Medical University Urumqi China; ^3^ Xinjiang Key Laboratory of Cardiac Electrophysiology and Remodeling The First Affiliated Hospital of Xinjiang Medical University Urumqi China; ^4^ Department of Cardiology Renmin Hospital of Wuhan University Wuhan Hubei China; ^5^ Section of Reproductive Endocrinology & Infertility Department of Obstetrics & Gynecology University of Oklahoma Health Sciences Center Oklahoma OK USA

**Keywords:** autoimmunity, gonadotropin‐releasing hormone receptor, inflammatory cytokine, insulin resistance, polycystic ovary syndrome

## Abstract

Polycystic ovary syndrome (PCOS), a metabolic and reproductive disease, is frequently associated with type 2 diabetes. We have demonstrated activating autoantibodies (AAb) directed toward the second extracellular loop (ECL2) of the gonadotropin‐releasing hormone receptor (GnRHR) are present in a significant subgroup of PCOS patients. It is unclear whether GnRHR‐AAb can induce peripheral tissue insulin resistance (IR) in animal models. Sixteen rats were divided equally into a GnRHR ECL2 peptide‐immunized group (IMM group) and a control group (CON group). Sera GnRHR‐AAb titer, luteinizing hormone (LH), and testosterone (T) were higher in IMM rats compared with CON rats. No significant difference in fasting blood glucose was observed between the two groups. However, the plasma glucose level at other time points of the IMM group was higher than that of the CON group during an intraperitoneal glucose tolerance test (IPGTT) and an insulin tolerance test (ITT) (*p* < 0.01). These data support the likelihood of the GnRHR‐AAb induction of glucose intolerance and IR. Compared with the CON group, the IMM group showed a significant increase in insulin‐stimulated phosphorylation of IRS‐1 (p‐IRS‐1 S636/639) and a decrease in insulin‐stimulated phosphorylation of Akt (p‐AKT S473). Expression of the glucose transport genes including GLUT‐2 in liver and GLUT‐4 in white adipose tissue and skeletal muscle was significantly decreased in IMM rats compared with the CON rats. Serum levels of proinflammatory cytokines (TNF‐α, IL‐1α, and IL‐18) were increased, while anti‐inflammatory cytokines (IL‐4 and IL‐10) were decreased in the IMM group. Taken together, elevated GnRHR‐AAb enhanced LH, hyperandrogenism, and inflammation. These changes are likely related to the observed peripheral tissue IR through the downregulation of the insulin‐stimulated IRS/PI3K/Akt/Glut signaling pathway.

## Graphical abstract

Proposed pathophysiological mechanisms of GnRHR‐AAb, hyperandrogenemia, inflammation, and insulin resistance in PCOS. GnRHR‐AAb promotes the release of LH from the pituitary gland. The presence of high concentrations of testosterone can be detected in the blood and elevated serum testosterone levels. These lead to the rebalancing (as shown by the tilt of the bar) of inflammatory factors in this disorder and thereby inducing insulin resistance. GnRHR‐AAb, GnRHR‐activating autoantibodies; LH, luteinizing hormone; IR, insulin resistance; TNF‐α, tumor necrosis factor‐alpha; IL‐1α, interleukin −1 alpha; IL‐4, interleukin 4; IL‐10, interleukin 10; IL‐18, interleukin 18.

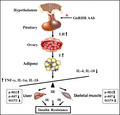

## 1 INTRODUCTION

Polycystic ovary syndrome (PCOS) is a common and complex endocrine disorder of women in their reproductive years, with prevalence between 5% and 10% (Azziz et al., 2004). PCOS is frequently associated with hyperandrogenism and an adverse metabolic profile including obesity and insulin resistance (Bremer, 2010). PCOS is characterized by a variable and erratic elevation of luteinizing hormone (LH) of unknown etiology. The hypothalamic/pituitary gonadotropin‐releasing hormone (GnRH) system is a key regulator of the reproductive system, triggering the synthesis and pulsatile release of LH from the pituitary (Christensen et al., 2012). The etiology of this heterogeneous disorder is unknown and previous attempts to identify an autoimmune pathophysiological target have been unsuccessful.

New research has shown that a significant subgroup of subjects with PCOS may have an autoimmune component contributing to their pathophysiology. Different autoantibodies previously have been documented in PCOS, for example, anti‐nuclear (ANA), anti‐thyroid, anti‐SM, anti‐histone, anti‐ovarian, and anti‐islet cell antibodies (Mobeen et al., 2016). We recently reported the presence of an activating autoantibody (AAb) directed toward the second extracellular loop (ECL2) of the GnRH receptor (GnRHR) in many subjects with confirmed PCOS. Purified IgG from some of these PCOS subjects with elevated GnRHR‐AAb demonstrated a dose‐dependent effect on GnRHR activation in GnRHR‐transfected cells; and this in vitro activity was suppressed by the GnRHR antagonist Cetrorelix (Kem et al., 2020). This discovery supported our hypothesis that GnRHR‐AAb was involved in the pathogenesis of PCOS.

Insulin resistance and compensatory hyperinsulinemia affect both obese and non‐obese women with PCOS (DeUgarte et al., 2005; Mathur et al., 2008). However, insulin resistance in lean PCOS patients was often ignored. In some cases, insulin resistance appears to be independent of obesity and related specifically to PCOS (Dunaif et al., 1987; Ovesen et al., 1993). Multiple studies have shown associations between insulin resistance and hyperandrogenism in human (Polderman et al., 1994) and animal models (Andrisse et al., 2018). Women with PCOS may have chronic low‐level inflammation (González, 2012). A recent study showed that excessive androgens caused adipocyte hypertrophy and increase inflammation, which was related to increased insulin resistance (Rui et al., 2001). However, it is still unclear whether androgen excess in PCOS promotes a state of inflammation.

In this study, we established an autoimmune rat model using a synthetic GnRHR ECL2 peptide to induce the production of GnRHR‐AAb and hyperandrogenemia. We evaluated the impact of this induced GnRHR‐AAb on insulin signaling and inflammatory status. The results demonstrated that immune and inflammatory elements serve as critical components in GnRHR‐AAb‐induced peripheral insulin resistance.

## 2 MATERIALS AND METHODS

### 2.1 Animals and experimental protocol

**FIGURE 1:**
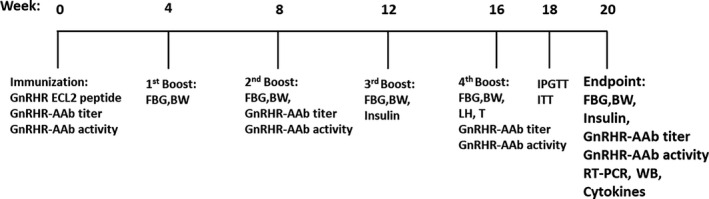
Immunization protocol performed on the rats. Sixteen rats were divided into two groups: an immune group (IMM group) and a control group (CON group). All rats in the IMM group were immunized with GnRHR ECL2 peptide at the beginning of the experiment and were boosted with peptide every 4 weeks. Rats were killed at the end of 20 weeks in which blood and tissue samples were collected for terminal studies. AAb, activating autoantibodies; GnRHR ECL2, second extracellular loop of gonadotropin‐releasing hormone receptor

Sixteen female Sprague Dawley rats (150–175 g) (Charles River) were divided into two equal groups: an immunized (IMM) group and a control (CON) group. Eight rats in the IMM group were immunized with 1 mg/kg of GnRHR ECL2 peptide (DSSGQTKVFSQC‐VTHCSFSQWWHQAFYN) (UniProtKB accession number P30968, LifeTein, Somerset, NJ) in 200 µl of complete Freund's adjuvant (F5881, Sigma), and subsequently boosted with the same peptide in incomplete Freund's adjuvant at 4, 8, 12, and 16 weeks. The CON group received saline in the same fashion. Blood from the saphenous vein was obtained from all rats for biochemical detection (Figure 1). These sera were always obtained just prior to the scheduled booster immunization for that date, thus reflecting serological values arising from the previous injections. All animal experimental procedures were reviewed and approved by the Institutional Animal Care Committee (IACUC 300837) and performed according to the American Veterinary Medical Association criteria.

### 2.2 Enzyme‐linked immunosorbent assay

GnRHR‐AAb titers in sera were determined by ELISA. Briefly, microtiter plates were coated with the GnRHR ECL2 peptide at 10 µg/ml in coating buffer. Sera were diluted in a series of concentrations from 1:4000 to 1:256,000 in dilution buffer. Alkaline phosphatase‐conjugated goat anti‐rat IgG (Jackson ImmunoResearch; RRID: AB_2338148; https://scicrunch.org/resolver/RRID:AB_2338148) and alkaline phosphatase substrate (S0942, Sigma) were used for antibody detection. Titers were determined as the highest dilution with an OD value of 0.30 at 30 min using a BioTek instrument (ELx808, BioTek Instrument).

LH (Enzo Life Sciences; RRID: AB_2848134; https://scicrunch.org/resolver/RRID:AB_2338134), total testosterone (T) (Enzo Life Sciences; RRID: AB_2848196; https://scicrunch.org/resolver/RRID:AB_2848196), and insulin (Thermo Fisher Scientific; RRID: AB_2848197; https://scicrunch.org/resolver/RRID:AB_2848197) concentrations were detected with ELISA kits. These assays were performed in duplicate for each serum sample. All experimental procedures were carried out according to the manufacturer's instructions. The intra‐assay coefficient of variation CV was 4.2% (*n* = 16) for LH, 6.3% (*n* = 16) for T, and 5.3% (*n* = 16) for insulin. Each given hormone assay was performed on the same plate to eliminate inter‐assay variance. The lower limit of sensitivity was 5.2 mIU/ml, 2.6 pg/ml, and 5 µIU/ml for LH, T, and insulin, respectively.

### 2.3 Cell‐based GnRHR assay

Serum activation of GnRHR in GnRHR‐NFAT‐bla CHO‐K1 cells (Thermo Fisher Scientific; RRID: CVCL_KB29; https://scicrunch.org/resolver/RRID:CVCL_KB29) was assessed using the GeneBLAzer FRET‐based β‐lactamase reporter assay (Invitrogen) according to the manufacturer's instructions. Briefly, cells were plated in 384‐well plates and incubated overnight. The individual serum samples were then added and incubated for 4 h. The β‐lactamase substrate CCF4‐AM (LiveBLAzer‐FRET B/G Loading Kit, Invitrogen) was then added and incubated at room temperature for 2 h in the dark. The plates were read using a fluorescence microplate reader (BioTek Synergy 2 Multi‐Detection Microplate Reader). All samples were assayed in triplicate. The intra‐assay coefficient of variation was 6.2% (*n* = 16) and the inter‐assay coefficient of variation was 4.6% (*n* = 16). Data were expressed as fold increase over buffer baseline to normalize the individual values. A fold increase value of 1.0 is activity equivalent to the buffer baseline value.

### 2.4 Body weight, fasting blood glucose, IPGTT, and ITT

Rat weights were measured using an electronic scale (A&D SJ‐12KHS, Old Will Knott Scales) at 0, 4, 8, 12, 16, and 20 weeks of immunization. For this procedure, each rat was fasted 16 h. Then 5 μl of blood (one drop) from the saphenous vein was transferred directly onto a glucose indicator strip for assay of blood glucose with a glucometer (ONETOUCH^@^Verio Flex, Lifescan). The IPGTT and ITT were performed at 18 weeks. For the IPGTT, the rats were fasted overnight (16 h) and then given an intraperitoneal injection of glucose (2 g/kg) and blood glucose was obtained before the glucose injection (0 min time point) and at 15, 30, 60, and 120 min thereafter. The rats were returned to their cage and monitored visually between each of these time points. Related area under curve (AUC), describing blood glucose levels in each rat after glucose loading, was then calculated (Jørgensen et al., 2017). For the ITT, insulin (0.75 U/kg) was injected by the IP route into rats after fasting for 6 h, and blood glucose was subsequently detected at the scheduled intervals (0, 15, 30, 60, 120 min).

### 2.5 In vivo insulin administration

Insulin was administrated to the rats prior to sacrifice in order to obtain “optimal insulin” signaling measurements as described below. At 20 weeks, the CON and IMM rats were fasted 6 h, followed by the administration of 0.5 U/kg body weight regular insulin (U‐100, Eli Lilly) or saline via IP injection 10 min before sacrifice. Liver and skeletal muscle tissues were obtained and frozen at −80°C for subsequent assays for insulin‐stimulated expression of IRS/PI3K/Akt/Glut signaling pathways.

### 2.6 Western blot analysis

Liver and skeletal muscle tissues (≤5 mg) were homogenized in 1 ml of RIPA lysis buffer. The protein concentrations were measured by a spectrophotometer (ND‐1000, NanoDrop, Marshall Scientific), and all samples were adjusted to the same protein concentrations. Samples were mixed with loading buffer, separated by SDS‐PAGE gel (Thermo Scientific), and transferred to a nitrocellulose membrane. TBS‐T containing 5% BSA was used to block the membrane before adding primary mouse anti‐rat antibodies p‐AKT (S473) (Cell Signaling; RRID: AB_329825; https://scicrunch.org/resolver/RRID:AB_329825) and p‐IRS‐1(S636/639) (Cell Signaling; RRID: AB_330339; https://scicrunch.org/resolver/RRID:AB_330339) at 1:1000 incubation. Membranes were washed three times with TBS‐T and secondary anti‐mouse IgG antibody (Cell Signaling; RRID: AB_2798581; https://scicrunch.org/resolver/RRID:AB_2798581) was diluted 1:1000 and used for incubation. Bands were visualized by enhanced chemiluminescence (Thermo Scientific), and the membranes were stripped and incubated with T‐AKT (Cell Signaling; RRID: AB_329827; https://scicrunch.org/resolver/RRID:AB_329827), T‐IRS‐1 (Cell Signaling; RRID: AB_330333; https://scicrunch.org/resolver/RRID:AB_330333), and β‐actin (Cell Signaling; AB_2223172; https://scicrunch.org/resolver/AB_2223172) to verify equal loading of proteins.

### 2.7 RT‐PCR analysis

RT‐PCR was performed to determine mRNA levels of glucose transport genes implicated in insulin resistance: Glut‐2 for liver tissue and Glu‐4 for white adipose tissue (WAT) and skeletal muscle tissue. Total RNA was isolated from the liver, WAT, and skeletal muscle using the TRIzol reagent (Bio‐Rad). The cDNA was synthesized using an Advantage RT for PCR Kit (Bio‐Rad). RT‐PCR amplification reactions were performed in a volume of 20 μl containing 1 μl of cDNA, 1 μl of forward/reverse primers, 10 μl of SYBP Green PCR master mix (Bio‐Rad), and 7 µl of nuclease‐free water by the iCycler iQ5 Q‐PCR machine (Bio‐Rad). All primers are shown in Table 1. All samples were run in triplicate reactions to confirm consistency in the amount of PCR products. The expression level of the genes of interest was corrected using GAPDH as a reference gene. The fold change was calculated according to the control group which was considered as 1.0.

**TABLE 1:** Primers used in RT‐PCR

Primer	Forward	Reverse
GLUT2	CTGGGTCTGCAATTTCATCA	CGTAAGGCCCGAGGAAGT
GLUT4	GCACAGCCAGGACATTGTTG	CCCCCTCAGCAGCGAGTGA
GAPDH	GTGGAGTCTACTGGCGTCTT	CCTGCTTCACCACCTTCTTG

### 2.8 Bio‐Plex Pro™ magnetic bead‐based assays

The levels of inflammatory cytokines including tumor necrosis factor (TNF‐α), interleukin (IL)‐1α, interleukin (IL)‐4, interleukin (IL)‐10, and interleukin (IL)‐18 were measured in sera from all rats. This was performed using a Bio‐Plex Pro™ magnetic bead‐based assays (Bio‐Rad, RRID: AB_2857368; https://scicrunch.org/resolver/RRID:AB_2857368) on the Bio‐Plex^®^ platform (Bio‐Rad) according to the manufacturer's instruction. Following optimization, samples were evaluated undiluted in a blinded manner. Bio‐Plex Manager™ software, version 6.0 was used for bead acquisition and analysis. The lower limit of sensitivity was 3 pg/ml, 1 pg/ml, 1 pg/ml, 5 pg/ml, and 4 pg/ml for TNF‐α, IL‐1α, IL‐4, IL‐10, and IL‐18, respectively.

### 2.9 Statistical analysis

Statistical analyses were conducted using SPSS v. 22 for Windows (IBM). The data are expressed as the mean ±SEM or SD, unless otherwise indicated. Comparison between two groups in LH, T, insulin signaling genes, and inflammatory cytokines were performed using the Student's *t* tests. Multiple group comparisons in body weight, blood glucose, insulin level, IPGTT, and ITT were performed using ANOVA with Bonferroni multiple comparison post hoc test. A significance level of *p* < 0.05 was used in all analyses.

## 3 RESULTS

### 3.1 GnRHR‐AAb production in IMM rats

**FIGURE 2:**
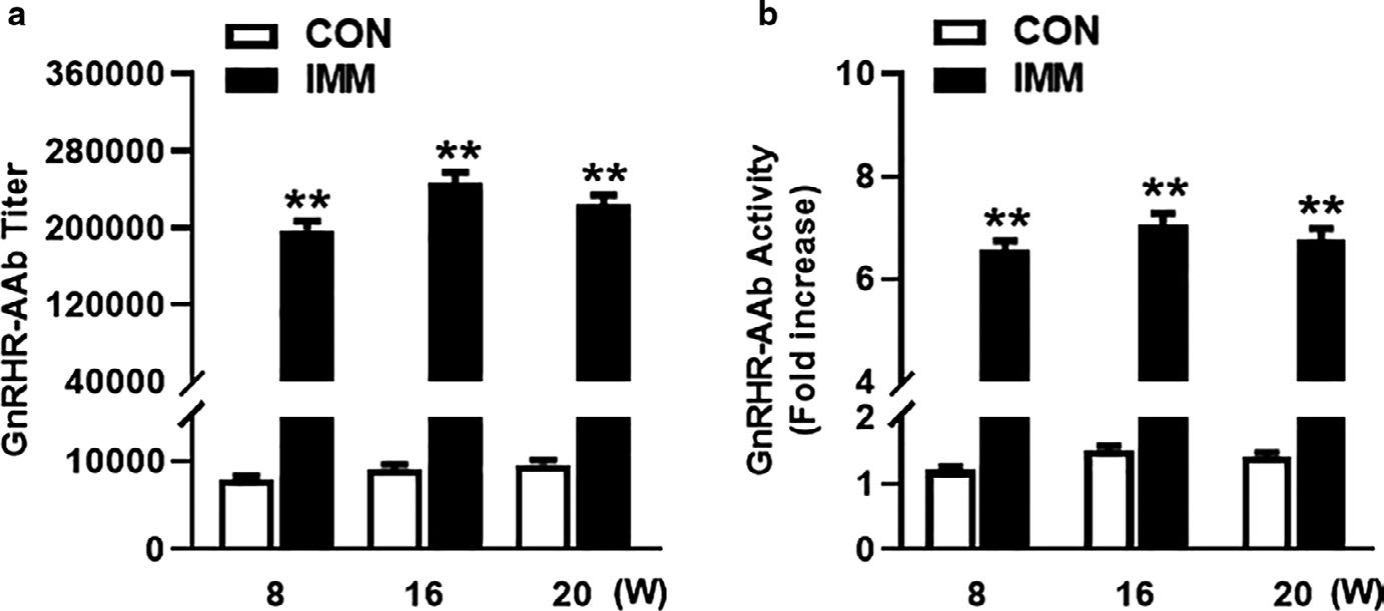
GnRHR antibody titer and activity in serum from IMM rats at week 0 and after 8, 16, and 20‐weeks of immunization and in CON rats. IMM rats developed high titers and activity of GnRHR‐AAb after immunization. Comparison between two groups was performed using Student's *t* test. **p < 0.01 versus CON, *n* = 8 (repeated measures ANOVA). CON, control; GnRHR‐AAb, GnRHR‐activating autoantibodies; IMM, immune

The titers (Figure 2a) and activity (Figure 2b) of GnRHR‐AAb at 0, 8, 16, and 20 weeks, respectively, were tested by ELISA and cell‐based bioassay to determine the immunogenicity of the ECL2 peptide. The IMM rats developed and maintained high antibody titers to GnRHR from 8 to 20 weeks (*p* < 0.01, *n* = 8). The GnRHR‐AAb activity for the IMM group was significantly greater than the CON group (*p* < 0.01, *n* = 8). These results demonstrated that antibodies capable of activating GnRHR were successfully induced at 8, 16, and 20 weeks after immunization.

### 3.2 Effects of GnRHR‐AAb on level of LH and testosterone in rats

**FIGURE 3:**
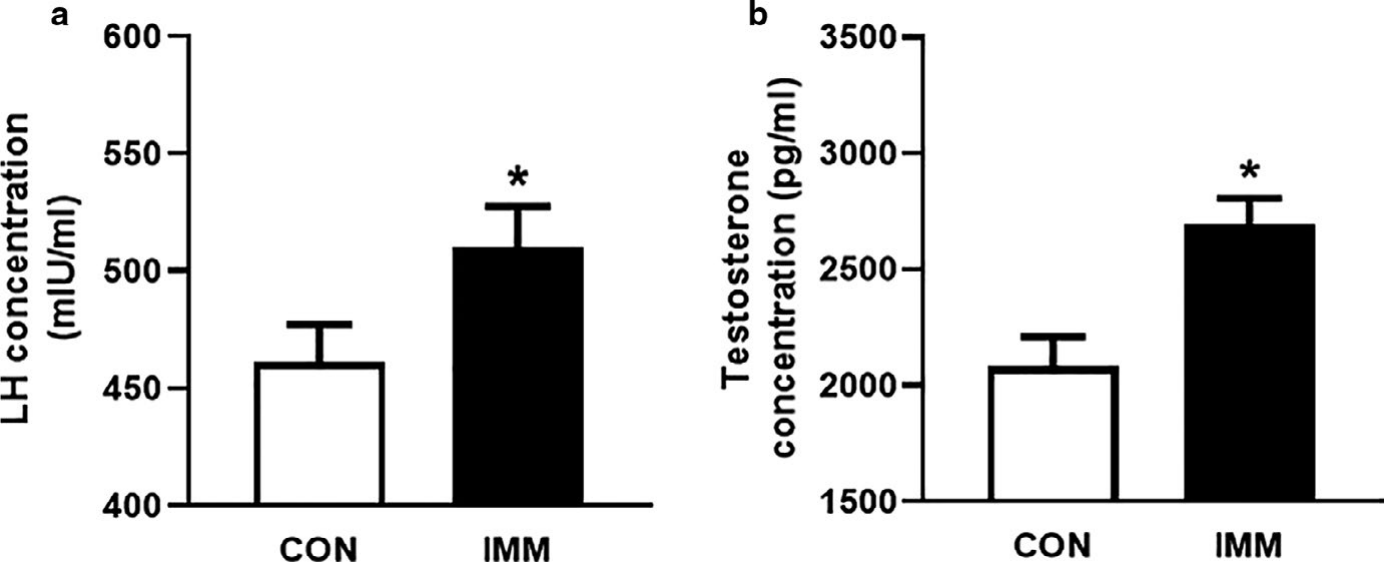
Effects of elevated GnRHR‐AAb on LH and T. Concentration of LH (a) and T (b) detected by ELISA in sera. IMM rats represented higher concentration of LH and T compared with CON rats after 16 weeks of immunization. Comparison between two groups was performed using Student's *t* test. *p < 0.05 versus CON, *n* = 8. CON, control; IMM, immune; LH, luteinizing hormone

The concentrations of LH (Figure 3a) and free T (Figure 3b) in both groups were detected with ELISA kits. Sera from rats at 16 weeks were used and diluted to 1:3. IMM rats displayed higher concentrations of LH and T compared with CON rats (*p* < 0.05, *n* = 8). These results demonstrated that GnRHR‐AAb stimulated the secretion of LH and led to hyperandrogenism in IMM rats.

### 3.3 Effects of GnRHR‐AAb on IPGTT, ITT, and body weight in rats

**FIGURE 4:**
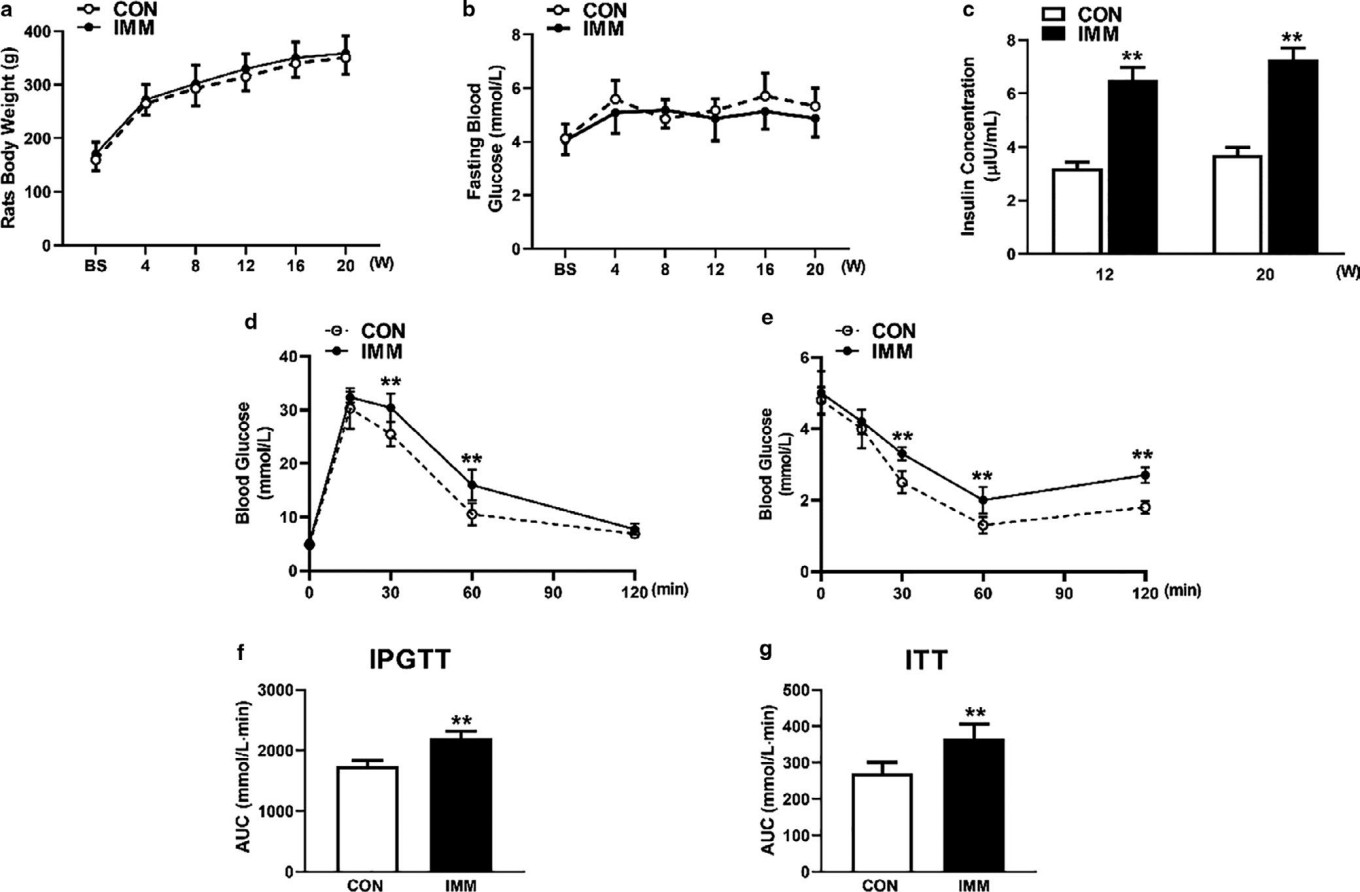
Effects of GnRHR‐AAb on glucose metabolism and insulin sensitivity. There was no significant difference in rat's body weight changes (a) or fasting blood glucose level (b) between the IMM group and the CON group. Insulin concentrations increased significantly compared with the CON group (c). Rats were injected at week 18 with 2 g/kg glucose at baseline (BS) and blood glucose level was tested at 15, 30, 60, and 120 min. Blood glucose levels of immune rats were higher at 30 and 60 min than those of CON rats (d). The incremental area under the 2‐h blood glucose response curve was compared and IMM rats displayed a significantly increased AUC (f). After insulin injection, the blood glucose concentration of the IMM rats was higher than that of the CON group at 30, 60, 120 min. (e). So as to the glucose area AUC during ITT (g). Multiple group comparisons were performed using ANOVA with Bonferroni multiple comparison post hoc test. **p < 0.01 versus CON. *n* = 8. AUC, area under curve; CON, control; IMM, immune; IPGTT, intraperitoneal glucose tolerance tests; ITT, insulin tolerance tests

During the experiment, all rats were measured for body weight (Figure 4a) and fasting blood glucose levels (Figure 4b). The body weight and fasting blood glucose of the IMM rats were not significantly different compared to CON rats (*p* > 0.05, *n* = 8). As the experiment proceeded, we observed that the fasting serum insulin in the IMM rats was significantly elevated compared with the CON rats at weeks 12 and 20 (*p* < 0.01, *n* = 8) (Figure 4c).

In order to test the effects of GnRHR‐AAb on metabolic parameters, we used an IPGTT to assess glucose tolerance. The glucose values for IMM rats at 30 and 60 min were significantly higher than those of CON rats with an apparent slower clearance (Figure 4d). The total AUC of blood glucose levels between 0 and 120 min was shown for the two groups in Figure 4f. The AUC of the IMM group was significantly higher than that of the CON group. These results demonstrated that GnRHR‐AAb could induce glucose intolerance in IMM rats.

We also used an ITT to evaluate insulin tolerance. After being injected with insulin, the expected decrease (decrement) in blood glucose levels in the IMM rats was significantly attenuated at 30, 60, and 120 min than that observed in the CON rats (Figure 4e). The glucose AUC during the ITT for the IMM rats was greater than that observed for the CON rats (*p* < 0.01, *n* = 8) (Figure 4g). All of these changes suggested that elevated GnRHR‐AAb induced glucose intolerance and insulin resistance, which was independent of the body weight and fasting blood glucose.

### 3.4 Effects of GnRHR‐AAb on the IRS/PI3K/Akt/Glut signaling pathway in the liver and skeletal muscle

**FIGURE 5:**
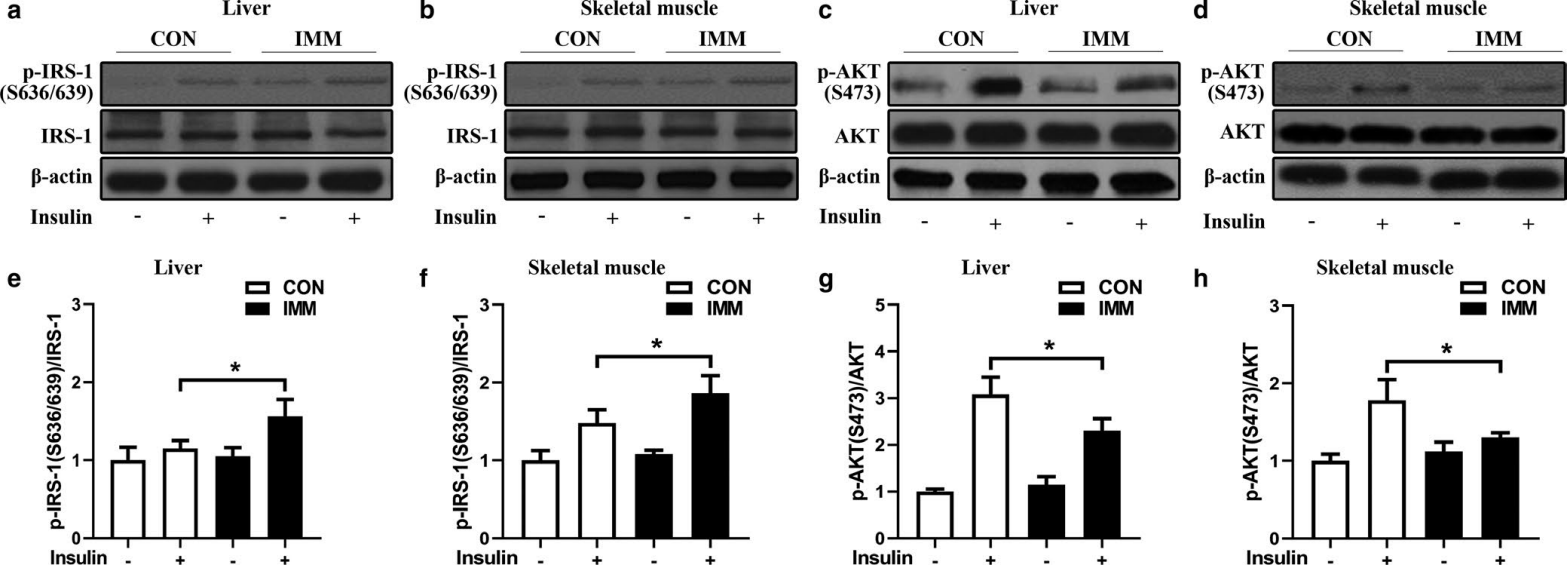
Effects of GnRHR‐AAb on expression levels of insulin signaling in peripheral tissues. Representative images of Western blot of ρ‐AKT (S473)/AKT and ρ‐IRS‐1(S 636/639)/IRS‐1 protein levels in liver tissue (a, e, c, g) and skeletal muscle (b, f, d, h). IMM rats displayed lower levels of insulin‐stimulated p‐AKT and higher levels of insulin‐stimulated p‐IRS‐1 in IMM rats compared to CON rats. β‐actin was used as a loading control. Comparison between two groups was performed using Student's *t* test. *p < 0.05 versus CON, *n* = 4. CON, control; IMM, immune; p‐AKT, phospho‐protein kinase B; p‐IRS‐1, phospho‐insulin receptor substrate 1

The expression levels of IRS/PI3K/Akt signaling pathway components in the rat liver, and skeletal muscle were determined by Western blot as shown in Figure 5 (a, e, c, g represents liver tissue, and b, f, d, h represents skeletal tissue). Compared with the CON group, the IMM group showed a significant increase in insulin‐stimulated serine phosphorylation of IRS‐1 (p‐IRS‐1 S636/639) and a decrease in insulin‐stimulated phosphorylation of Akt (p‐AKT S473), suggesting a GnRHR‐AAb‐mediated impairment of insulin signaling through upregulating insulin‐stimulated p‐IRS‐1(S636/639) and downregulating insulin‐stimulated p‐Akt473 in IMM liver and skeletal muscle.

**FIGURE 6:**
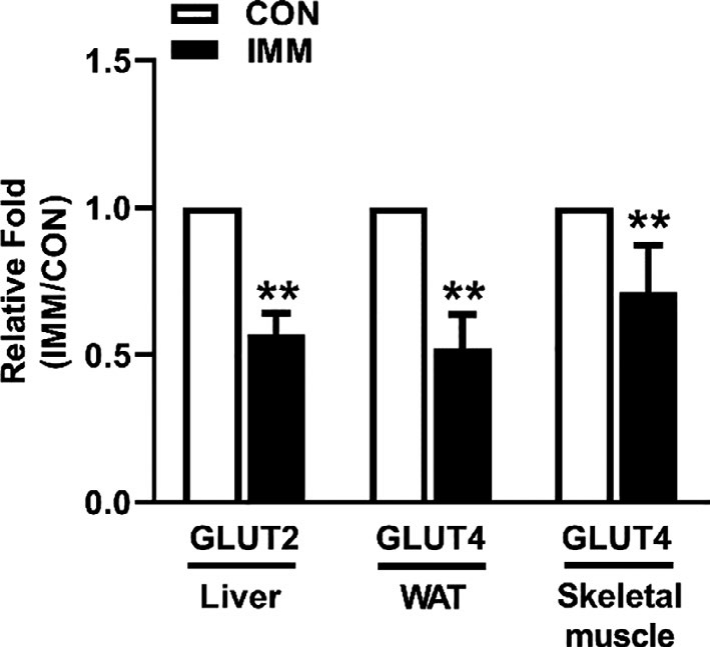
Effects of GnRHR‐AAb on glucose transporter genes in peripheral tissues. The mRNA expression of glucose transporter genes in the liver (GLUT‐2), white adipose tissue (GLUT‐4), and skeletal muscle (GLUT‐4) were significantly lowered following GnRHR‐ECL2 immunization compared with the CON rats. Comparison between two groups was performed using Student's *t* test. **p < 0.01 versus CON, *n* = 8. CON, control; GLUT, glucose transporter; IMM, immune

The mRNA expressions of Glut components in the rat liver, WAT, and skeletal muscle were determined by RT‐PCR as shown in Figure 6. Representative glucose transport‐related genes included liver GLUT‐2, WAT GLUT‐4, and skeletal muscle GLUT‐4. These were significantly decreased in IMM rats compared with the CON rats, suggesting that GnRHR‐AAb led to peripheral tissue insulin resistance by downregulating the insulin‐stimulated IRS/PI3K/Akt/Glut signaling pathway. Expression of the GAPDH reference gene did not change across the two groups of rats.

### 3.5 Effects of GnRHR‐AAb on proinflammatory and anti‐proinflammatory cytokines

**FIGURE 7:**
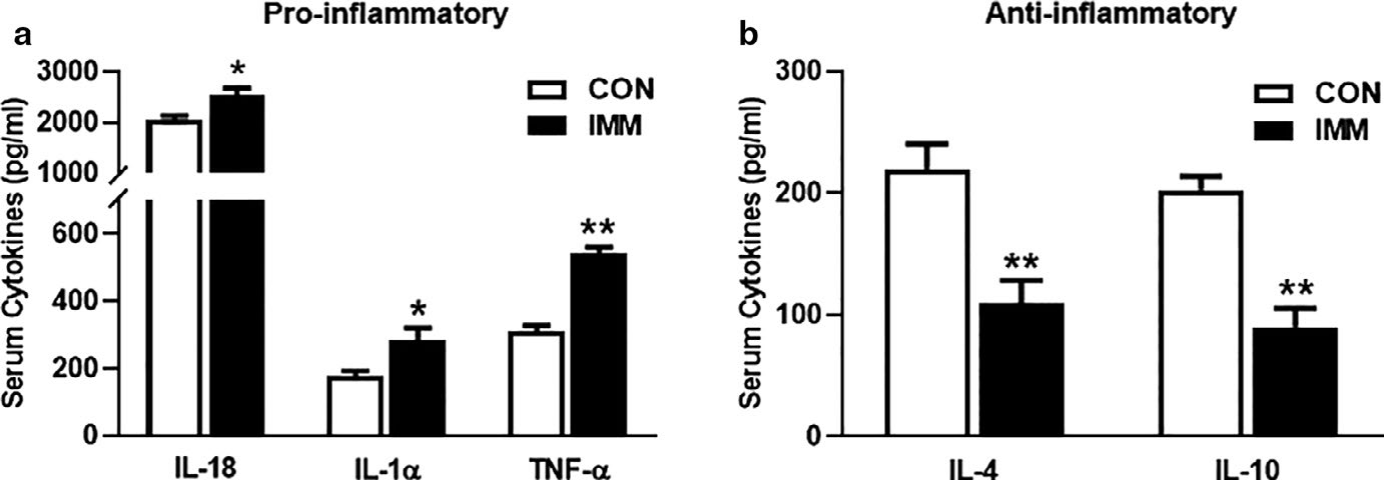
Effects of GnRHR‐AAb on levels of TNF‐α, IL‐1α, IL‐4, IL‐10, and IL‐18. The concentrations of the pro‐inflammatory cytokines (TNF‐α, IL‐1α, IL‐18) were significantly increased in the sera of GnRHR immunized rats when compared with the CON rats (a). IMM rats displayed significantly reduced circulating anti‐proinflammatory proteins (IL‐4, IL‐10) when compared with normal CON rats (b). Comparison between two groups was performed using Student's *t* test. *p < 0.05 versus CON, **p < 0.01 versus CON, *n* = 8. CON, control; IL‐10, interleukin 10; IL‐18, interleukin 18; IL‐1α, interleukin‐1 alpha; IL‐4, interleukin 4; IMM, immune; TNF‐α, tumor necrosis factor‐alpha

The effects of GnRHR‐AAb on TNF‐α, IL‐1α, IL‐4, IL‐10, and IL‐18 are illustrated in Figure 7. The concentration of the proinflammatory cytokines (TNF‐α, IL‐1α, IL‐18) was significantly increased in the sera of the IMM rats compared with the CON rats (Figure 7a). IMM rats also demonstrated significantly reduced anti‐proinflammatory cytokines (IL‐4, IL‐10) when compared with CON rats (*n* = 8) (Figure 7b). This indicated that inflammation may play an important role in the insulin resistance induced by the GnRHR‐AAb.

## 4 DISCUSSION

**FIGURE 8:**
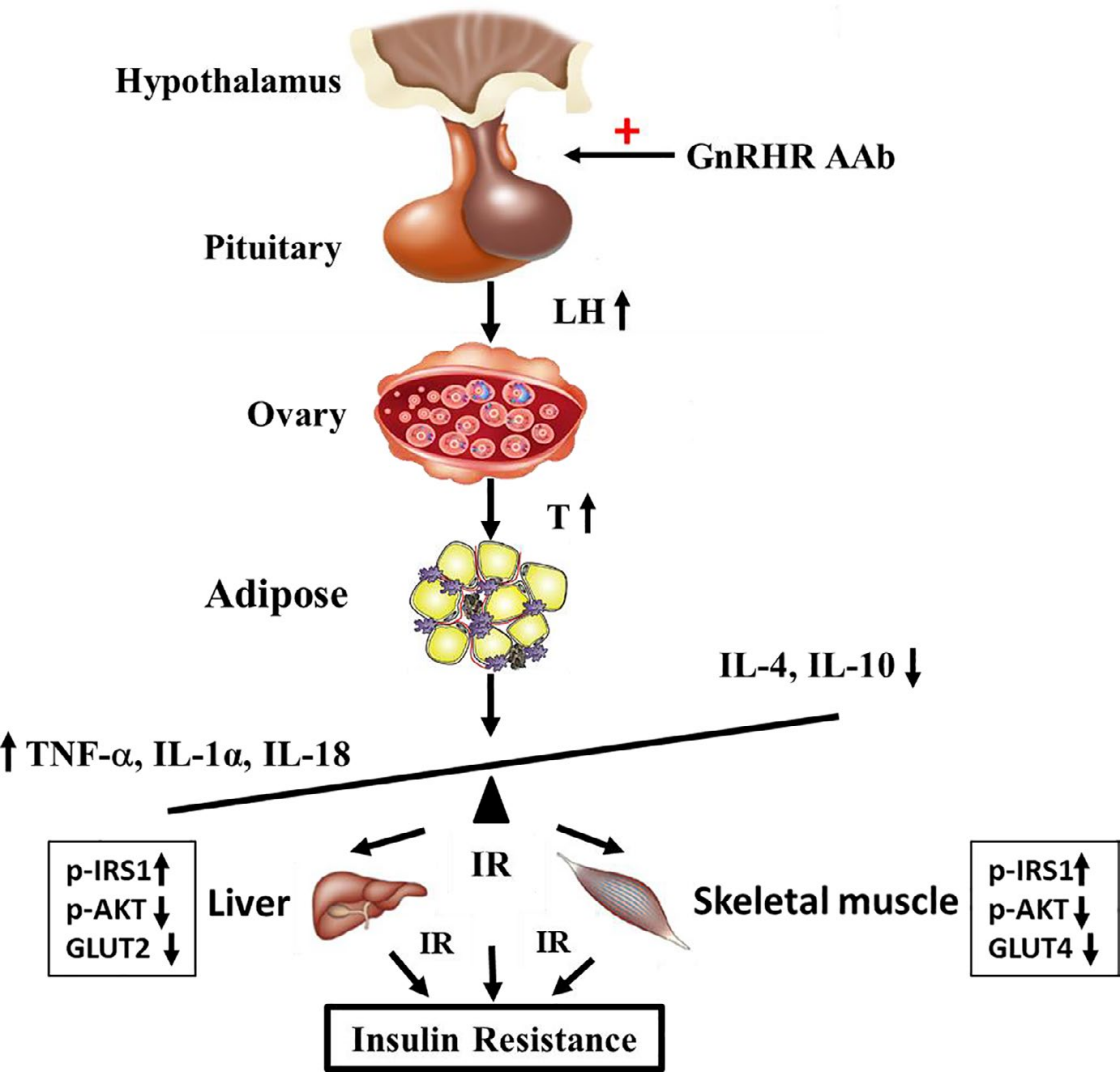
Proposed pathophysiological mechanisms of GnRHR‐AAb, hyperandrogenemia, inflammation, and insulin resistance in PCOS. GnRHR‐AAb promotes the release of LH from the pituitary gland. The presence of high concentrations of testosterone can be detected in the blood and elevated serum testosterone levels. These lead to the rebalancing (as shown by the tilt of the bar) of inflammatory factors in this disorder and thereby inducing insulin resistance. GnRHR‐AAb, GnRHR‐activating autoantibodies; IL‐10, interleukin 10; IL‐18, interleukin 18; IL‐1α, interleukin‐1 alpha; IL‐4, interleukin 4; IR, insulin resistance; LH, luteinizing hormone; TNF‐α, tumor necrosis factor‐alpha

We have developed an autoimmune rat model using a specific synthetic GnRHR ECL2 peptide to induce significant GnRHR‐AAb activity. Serum LH and T concentrations were elevated in this rat model and compatible with those observed in humans with PCOS. Even after a relatively short period of GnRHR‐AAb stimulation (20 w), the IMM rats showed glucose intolerance and insulin resistance independent of body weight and fasting blood glucose. The increased GnRHR‐AAb and testosterone were associated with the induced insulin resistance and impaired insulin response to a glucose challenge. There was evidence for sub‐normal IRS‐1‐PI3K‐Akt‐Glut signaling and increased levels of specific inflammatory cytokines. Our results support the concept that the selective autoantibody activation of the GRHR and the consequent increase in androgens and chronic inflammation are important mechanisms relating to insulin resistance (Figure 8).

We evaluated glucose disposal and insulin sensitivity through IPGTT and ITT tests. Our results demonstrated the blood glucose levels at 30 min and 60 min in the IPGTT test were increased and the AUC blood glucose concentration during IPGTT was also increased in IMM rats. Thus, it provides evidence that GnRHR‐AAb reduced the efficiency of glucose removal. The insulin levels were significantly increased and an ITT estimating the peripheral insulin resistance was significantly higher in IMM rats than in the CON group. These differences were observed at a time when there had been no change in their body weight or in their fasting blood glucose compared with the CON rats. These data suggest that IMM rats developed insulin resistance prior to developing overt obesity and post‐glucose‐challenge hyperglycemia. These changes have been observed in PCOS subjects who have had much longer periods, often several years, of abnormal LH and T. A recent study of young PCOS humans demonstrated these early manifestations of insulin resistance prior to overt hyperglycemia (Fulghesu et al., 2010).

The apparent interactions between GnRHR‐AAb and the insulin signaling pathway are yet to be fully understood. Andrisse et al. (2018) established a PCOS mouse model by the administration of low dose dihydrotestosterone (DHT) and found that energy storage tissues displayed differential effects on the insulin signaling pathway. The liver and WAT displayed lower mRNA and protein expression of insulin signaling intermediates, while skeletal muscles exhibited normal levels. We also found gene expression of GLUT‐2 in liver, GLUT‐4 in WAT, and skeletal muscle were significantly decreased in IMM rats compared with the CON rats. We additionally observed the IMM rats showed a significant increase in insulin‐stimulated serine phosphorylation of IRS‐1 (p‐IRS‐1 S636/639) and a decrease in insulin‐stimulated phosphorylation of Akt (p‐AKT S473). We hypothesize that GnRHR‐AAb induces LH secretion resulting in an increase in the synthesis and secretion of androgens and thereby interrupting insulin signaling pathways (IRS‐1‐PI3K‐Akt‐Glut) in peripheral tissues. This would support the concept that GnRHR‐AAb‐induced hyperandrogenemia may be a significant component for the induction of insulin resistance.

We observed a reciprocal rise in the inflammatory response in the IMM group. Compared with the CON group, the levels of pro‐inflammatory cytokines (TNF‐α, IL‐1α, and IL‐18) were significantly increased, while the levels of the anti‐inflammatory cytokines (IL‐4 and IL‐10) were significantly decreased in the IMM group. Our findings were consistent with previous studies demonstrating increased proinflammatory and decreased anti‐inflammatory cytokines in PCOS patients (Amato et al., 2003; Duleba & Dokras, 2012; Heinrich et al., 1990; Makedos et al., 2011; Okamura et al., 1998; Stephens et al., 1992; Vural et al., 2010; Yang et al., 2011). Our experiment indicated that GnRHR‐AAb may induce an imbalance between pro‐ and anti‐inflammatory cytokines and thereby enhance peripheral insulin resistance. In a recent report, Qi et al. showed that serum IL‐22 level was also reduced in a mouse model of PCOS with gut microbiota modification, and administration of IL‐22 improved the PCOS phenotype, suggesting an important role of this anti‐inflammatory cytokine in gut microbiota‐induced insulin resistance and ovarian dysfunction in PCOS (Qi et al., 2019). It will be of interest to evaluate this in our rat PCOS model. Previous studies have implied that androgen, adipose tissue, and inflammatory factors are closely linked (Eisner et al., 2003; Solano et al., 2011). It is still unclear whether androgen excess in PCOS directly promotes a state of inflammation. Androgens cause adipocyte hypertrophy and this could explain their involvement in the development of chronic low‐grade inflammation (Spritzer et al., 2015). Our data highlight the potential importance of GnRHR‐AAb in humans and in an animal model for further investigation of inflammation and insulin resistance.

We have reported that PCOS serum IgG accentuates the agonist effect of GnRH in the GnRHR bioassay (Kem et al., 2020). AAb targeting G protein‐coupled receptors such as the GnRHR‐AAb do not desensitize their target receptor activity in contrast to the receptor's normal ligand LHRH (Kem et al., 2020). These AAb, when present, will not suppress GnRHR activity and may enhance it through both orthosteric and/or allosteric activity (Kem et al., 2020). This effect was observed during this study with the elevation of both GnRHR‐AAb, LH, and T under the influence of the GnRHR‐AAbs.

Currently, PCOS animal models are mainly divided into three types: genetic related (Houten & Visser, 2014), androgenic (Houten et al., 2012; Liu et al., 2000; Roland et al., 2010; Solano et al., 2011), and pharmacological‐induced models (Chaudhari et al., 2018; Coutinho & Kauffman, 2019; Kauffman et al., 2015). We are the first to develop a rat model of autoimmune PCOS using GnRHR ECL2 peptide immunization. These GnRHR IMM rats appear to share several features, including insulin resistance, with human PCOS.

In summary, we have provided evidence that GnRHR‐AAb induces insulin resistance in peripheral tissue early during the development of several manifestations of a PCOS‐like phenotype. Our studies provide new knowledge regarding the possible etiology of insulin resistance in some forms of PCOS and associated metabolic conditions, as well as a pathway for the development of novel effective treatments.

## CONFLICT OF INTEREST

The authors declare no conflicts of interest.

## AUTHOR CONTRIBUTIONS

Hongliang Li and Gege Zhang contributed equally to this work. Drs. Gege Zhang, Yankai Guo, and Jielin Deng served as Research Scholars in our laboratory during this study.
